# Fault Diagnosis and Maintenance Countermeasures of Transverse Drainage Pipe in Subway Tunnel Based on Fault Tree Analysis

**DOI:** 10.3390/ijerph192315471

**Published:** 2022-11-22

**Authors:** Shiyang Liu, Xuefu Zhang

**Affiliations:** 1 College of Civil Engineering, Chongqing Jiaotong University, Chongqing 400074, China; 2 State Key Laboratory of Mountain Bridge and Tunnel Engineering, Chongqing Jiaotong University, Chongqing 400074, China

**Keywords:** FTA, subway tunnel, transverse drainage pipe, crystalline blockage, drainage system maintenance

## Abstract

Transverse drainage pipe, one of the main channels of groundwater behind the lining of subway tunnels, plays an important role in the safety and stability of the tunnel lining structure. For the problem of blocked transverse drainage pipe in a subway tunnel, a fault tree model of blocked transverse drainage pipe in Chongqing subway tunnel was constructed in this paper, the quantitative and qualitative analysis of fault tree was conducted, and countermeasures for maintenance of transverse drainage pipe were proposed. The study finds that, (1) the chemical type of groundwater was mainly CaHCO_3_; most of the groundwater is strongly alkaline with pH greater than 8; the groundwater temperature is 20 ± 3 °C; (2) the basic events of blocked transverse drainage pipe have 3 minimum cut sets, and the basic events concrete slurry enters the drainage pipe; groundwater temperature, groundwater pH value, and concentration of anions and cations in groundwater were the main fault factors of blocked transverse drainage pipe; (3) preventive maintenance of transverse drainage pipe during tunnel construction includes construction quality control of drainage pipe and application of anti-crystallized blocking drainage pipe; preventive maintenance of transverse drainage pipe during tunnel operation includes monitoring of groundwater ion concentration, pH, and temperature; and maintenance treatment of transverse drainage pipe during tunnel operation includes physical treatment techniques, such as ultrasonic resonance, and chemical treatment techniques, such as acid-base neutralization reaction. The results of the study have certain guiding significance for the design, construction, and operation of transverse drainage pipe in subway tunnels.

## 1. Introduction

**Figure 1:**
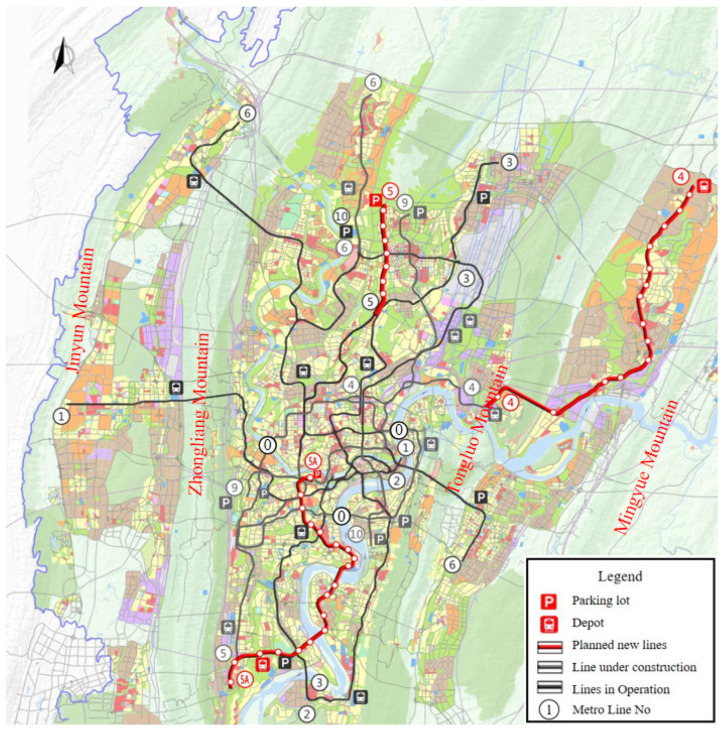
Schematic diagram of Chongqing urban rail transit phase III construction plan (2018–2023).

**Figure 2:**
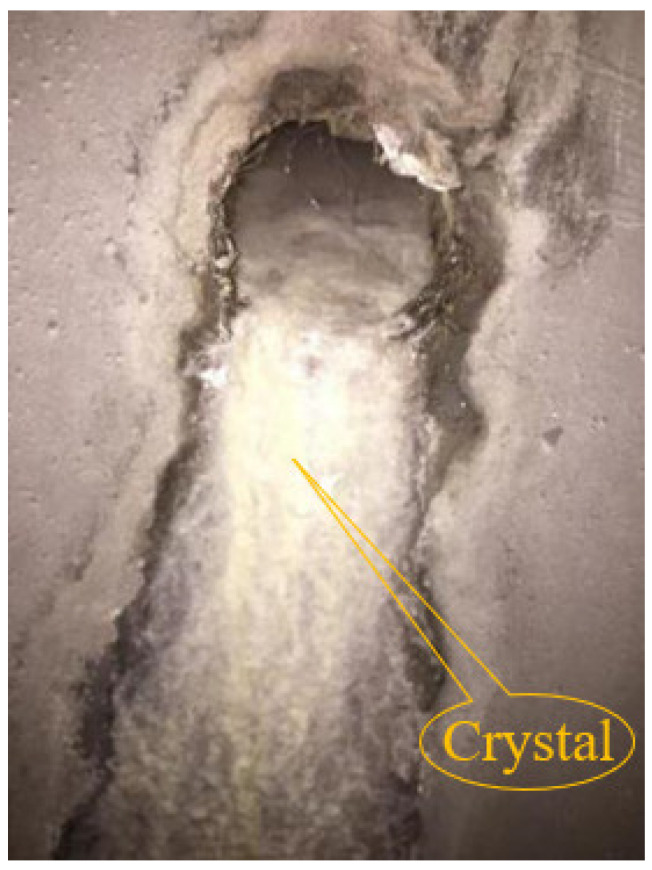
Crystal blockage of drainage pipe of Chongqing Metro Tunnel.

As of June 2022, 277 urban rail transit lines with 9067 km of operational mileage are in operation nationwide. Among them, Chongqing has 10 lines in operation, with an operating mileage of 434.6 km, ranking the 7th in China. After the completion of Phase III Chongqing Construction Planning (Figure 1), the traffic mileage of Chongqing’s rail transit is expected to exceed 500 km. Four major mountains, namely Mingyue, Tongluo, Zhongliang, and Jinyun, lie from east to west in the main urban area (Figure 1). As an important carrier, the tunnel is indispensable in the urban rail transit infrastructure. As an underground building (structure), the tunnel is inevitably affected by surrounding groundwater in the operation process. In recent years, the disease of tunnel drains has become increasingly noticeable, especially the crystalline blockage in transverse drainage pipe (Figure 2). After the drainage pipe is blocked, the groundwater behind the lining cannot be discharged in time, and the water pressure gradually increases, which seriously threatens the safety of the tunnel lining structure.

Fault tree analysis (FTA) can identify the cause of system failure, and find the best way to reduce risks. FTA has been widely used in the civil engineering field. In terms of construction, Zhou [1] established a fault tree for slope excavation construction, and identified various failure modes causing system failure in the foundation pit of slope excavation construction. Xu et al. [2] built a fuzzy fault tree analysis model for tunnel fires based on system safety engineering theory and fire science. Zhu et al. [3] applied FTA to the reliability simulation analysis of urban rail transit—the ATS subsystem. Ardeshir et al. [4] analyzed the risk assessment of the Dasht-e Zahab water-conveyance tunnel project based on a fuzzy failure tree. Hyun et al. [5] constructed a shield TBM tunnel failure tree set by taking geology, design, and construction (management) as risk factors. Lin et al. [6] established a fault tree for the instability of the Maluanshan Tunnel portal. Zhang et al. [7] established an incident tree model of the Nanjing Yangtze River Tunnel after the closure of the Nanjing Yangtze River Bridge. Wei [8] used FTA and AHP to evaluate the instability risk of the foundation pit enclosure of tunnel shafts in subway construction. Mottahedi and Ataei [9] used a coal rock burst as the top event of the fault tree to conduct a coal rock burst probability analysis. By integrating the T-S fuzzy fault tree and the Bayesian network, Chen et al. [10] proposed a method to evaluate the possibility of tunnel collapse when constructed by drilling and blasting methods. Zhao et al. [11] established a fault tree of urban rail transit operation. Yang and Deng [12] used the WBS-RBS method to identify the construction risk of the Qingpingchuan tunnel.

In terms of tunnel diseases, Pan et al. [13] established an evaluation index system and AHP model for water leakage hazards in tunnels. Chen and Yang [14] applied FTA, a risk analysis theory, to analyze and determine the risk of water leakage in highway tunnel structures. Liu et al. [15] evaluated the negative effects of groundwater environments in the Geleshan Tunnel of the Chongqing-Huaihua Railway by using the evaluation index system of negative effects of groundwater environments in a karst tunnel. Wang et al. [16] analyzed the impact factors of urban drainage facilities’ safety risks from three aspects: the drainage facility’s own impact factors, subway construction impact factors, and environmental risk factors, and formed an index system of impact factors of urban drainage facilities. Based on statistical analysis of the measured data of lining water pressure, Wang et al. [17] established a comprehensive evaluation system of tunnel water pressure, and made suggestions on the selection of tunnel waterproof and drainage patterns, based on the fuzzy evaluation results of water pressure. By comprehensively taking into account the impact of construction groundwater discharge on the change characteristics of the groundwater level and impact radius, as well as geography, geology, hydrogeology, tunnel engineering, and other related factors, Bai et al. [18] built a hierarchical model for comprehensive evaluation of the impact of tunnel groundwater discharge on ecological environments. Ding et al. [19] established a fault tree for the diseases of the Shanghai metro tunnel. Luo et al. [20] proposed an evaluation system of waterproof and drainage patterns of subway tunnels based on the AHP method, and established an AHP model to realize the quantification of waterproof and drainage type selection. Huang et al. [21] divided the quality evaluation levels and methods of waterproof and drainage system engineering of highway tunnels, and established the corresponding inspection and evaluation index system. Huang et al. [22] established a fault tree for the causes of functional diseases of the civil structure of operating tunnels. Ma and Deng [23] established an evaluation model and evaluation system of waterproof and drainage systems of highway tunnels using a fuzzy comprehensive evaluation method. Nie and Rao [24] constructed a fuzzy evaluation system for the crystalline blockage risk of tunnel drainage systems by a multi-level fuzzy comprehensive evaluation method.

FTA has been widely used in the engineering field, and there are some applications in tunnel engineering, such as tunnel water leakage disease, the safety and stability of the main structure, construction quality inspection, etc. Some scholars have built risk fuzzy evaluation systems for the environmental effects of groundwater, lining water pressure, and crystalline blockage of drainage systems in highway tunnel construction, but most of these studies focused on how to establish risk assessment models without putting forward quantitative or qualitative management and maintenance countermeasures in how to select the index system for the risk assessment model and how to guide the tunnel operation and management with the assessment results.

In view of the shortcomings of the existing studies and practical problems of engineering, the thesis constructs a fault tree model for the blockage of transverse drainage pipe in the Chongqing subway tunnel, carries out quantitative and qualitative analysis of the fault tree, and proposes countermeasures for the maintenance of transverse drainage pipes.

## 2. Sampling

**Figure 3:**
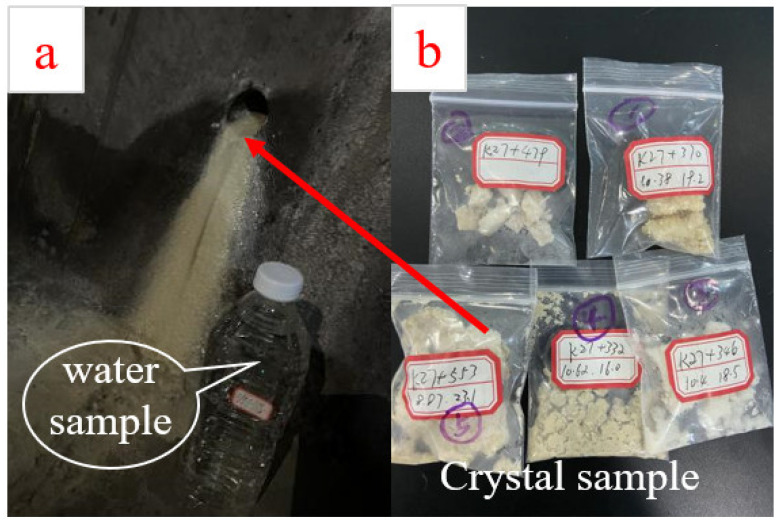
Field sampling. (**a**) water sample; (**b**) crystal sample.

In order to obtain the blockage of transverse drainage pipe in the Chongqing subway tunnel, more than 200 transverse drainage pipes were observed. At the typical blocking location of transverse drainage pipes, 1500 mL of groundwater samples were collected in standard water sample bottles (Figure 3a) and crystalline samples were collected with sealed bags (Figure 3b), while groundwater temperature and pH were tested with portable testing equipment. The collected groundwater and crystalline samples were commissioned to a professional institution for testing according to national standards, and the relationship between groundwater environment and transverse drainage pipe blockage was further analyzed based on the test results. A total of 7 groundwater samples and 5 crystalline samples were collected for testing. The detailed sampling location and on-site groundwater temperature and pH are provided in Table 1.

## 3. Construction and Analysis of a Fault Tree Model for Blocked Transverse Drainage Pipe in Subway Tunnels

### 3.1. Selection of Evaluation Methods for Lateral Drain Pipe Blockage

At present, there is no research on the evaluation of the drainage system of subway tunnels, most of which focus on the evaluation of the leakage disease of the tunnel lining structure, and the drainage system is only a part of it. As mentioned above, the fault tree analysis (FTA) method is used to analyze water leakage, structural cracking, structural deformation exceeding the standard, and segment joint opening of the tunnel structure; the fuzzy comprehensive evaluation method is used to study the crystal blockage risk of the tunnel drainage system, and the analytic hierarchy process is used to study the drainage status of the tunnel lining drainage system unit. These three methods have their own advantages and disadvantages.

Fault tree analysis. Advantages: (1) the causal relationship of the fault tree is clear and visual, (2) according to the frequency data of failure of each basic event, determine the impact of each basic event on the occurrence of the accident—structural importance, (3) it can be used for qualitative analysis, quantitative analysis, and systematic evaluation. Disadvantages: (1) FTA analysis of the accident cause is a strong point, but the possibility that the accident is the result of the identified cause is speculated to be a weak point, (2) FTA analysis is aimed at a specific accident rather than a process or equipment system, so it is local, (3) it is required that the analyst be very familiar with the object system being analyzed and be able to apply the analysis method accurately and skillfully; the fault tree prepared by different analysts and the analysis results are often different, (4) for complex systems, there are many steps to prepare the fault tree; the fault tree prepared is also large, and the calculation is complex, which brings difficulties to qualitative and quantitative analysis, and (5) to conduct quantitative analysis on the system, the probability of all basic events must be determined in advance, otherwise quantitative analysis cannot be conducted.

Fuzzy comprehensive evaluation analysis method. Advantages: (1) fuzzy evaluation deals with fuzzy evaluation objects through accurate digital means, and can make a more scientific, reasonable, and practical quantitative evaluation on the data containing fuzzy information, (2) the evaluation result is a vector rather than a point value; it contains rich information, which can not only accurately depict the evaluated object, but also further the process to obtain reference information. Disadvantages: (1) the calculation is complex and the determination of index weight vector is subjective, and (2) when the index set U is large—that is, when the number of index sets is large—under the constraint that the sum of the weight vectors is 1, the weight coefficient of the relative membership degree is often small, and the weight vector does not match the fuzzy matrix R, resulting in a super fuzzy phenomenon with poor resolution, which makes it impossible to distinguish who has a higher membership degree, or even leads to the failure of the evaluation; in this case, the hierarchical fuzzy evaluation method can be used to improve results.

Analytic Hierarchy Process. Advantages: (1) a systematic analysis method, which takes the research object as a system and makes decisions according to the decomposition, comparative judgment, and comprehensive thinking mode, has become an important tool for system analysis developed after mechanism and statistical analyses; (2) simple and practical decision-making methods, not only pursue advanced mathematics, but also do not one-sidedly focus on behavior, logic, and reasoning, but organically combine qualitative methods with quantitative methods, and (3) the quantitative data information required is less, which is mainly based on the evaluator’s understanding of the nature and elements of the evaluation problem, and focuses more on qualitative analysis and judgment than general quantitative methods. Disadvantages: (1) it cannot provide new solutions for decision-making, and the role of AHP is to select the better alternative, (2) there are few quantitative datum and many qualitative components, which are not convincing, (3) when there are too many indicators, the data statistics are large and the weight is difficult to determine, and (4) the exact calculation of eigenvalues and eigenvectors is complex.

It can be seen from the comparative analysis of several commonly used methods that the fault tree analysis method is simple and clear, only needing to determine the target object (top event), and then determine the basic events that cause the top event in turn. The analysis process is highly logical; it can provide targeted prevention or treatment measures according to the analysis results, which is convenient for staff to quickly master. The fuzzy comprehensive evaluation method and the analytic hierarchy process are complex and subjective. The analysis results cannot provide management or treatment plan for staff. Therefore, the fault tree analysis method is selected for the fault diagnosis analysis of the subway tunnel drainage system, and the prevention or treatment scheme is proposed based on the analysis results.

Fault tree analysis is a deductive failure analysis method from top to bottom. It uses Boolean logic to combine low order events to analyze unwanted states in the system. Fault tree analysis is mainly used in the fields of safety and reliability engineering to understand the causes of system failure and find the best way to reduce risk, or to confirm the occurrence rate of a safety accident or specific system failure.

### 3.2. Analysis of Groundwater Ion Concentration

The seven groundwater samples were commissioned to the Institute of Nuclear Industry 280 for testing. The test results of ion concentration in each groundwater sample are provided in Table 2.

As can be observed in Table 2, the Ca^2+^ concentration in groundwater ranged from 35 to 80 mg/L, the Mg^2+^ concentrations were all lower than 20 mg/L, the total groundwater hardness ranged from 140 to 270 mg/L, and the HCO_3_^−^ concentration ranged from 100 to 220 mg/L. Among the seven groundwater samples, one groundwater sample (K27 + 428) had a HCO_3_^−^ concentration of 0, but CO_3_^2−^, OH^−^ not 0, which was in contrast with that of the remaining six groundwater samples. The groundwater of this groundwater sample was strongly alkaline (pH greater than 10, greater than that of the remaining six groundwater samples). This was because the chemical reaction between HCO_3_^−^ and OH^−^ generated CO_3_^2−^, and the excess OH^−^ was in a free state. The free CO_2_ concentration of this groundwater sample was 0, while the remaining six groundwater samples all contained free CO_2_, indicating that the CO_2_ of this groundwater sample was dissolved in water and involved in the crystallization reaction.

Moreover, the pH of groundwater in the field test was different from that of the indoor test. The reasons for analysis are mainly: (1) the groundwater in the field test was in a flowing state while basically in a static state in the indoor test, and (2) the groundwater has been stored in a sealed environment in the indoor test, and the concentration of anions and cations in the groundwater has changed. As can be observed in Table 2, the temperature of groundwater in the field test was between 17 and 23 °C, basically within the range of 20 ± 3 °C. Most of the groundwater at the crystallization site of transverse drainage pipe was strongly alkaline. Although the temperature of groundwater samples (K27 + 346 and K27 + 332) was lower than that of the remaining groundwater samples, their pH was significantly higher than that of the remaining groundwater samples, indicating that pH had a greater impact on the generation of crystal than temperature.

### 3.3. Test and Analysis of Crystalline Composition

**Figure 4:**
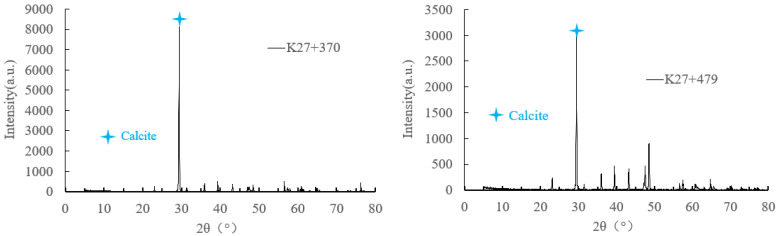

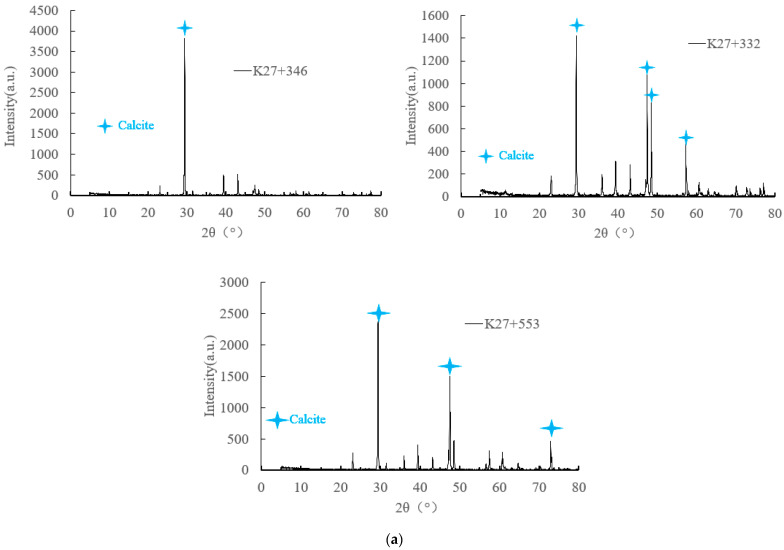

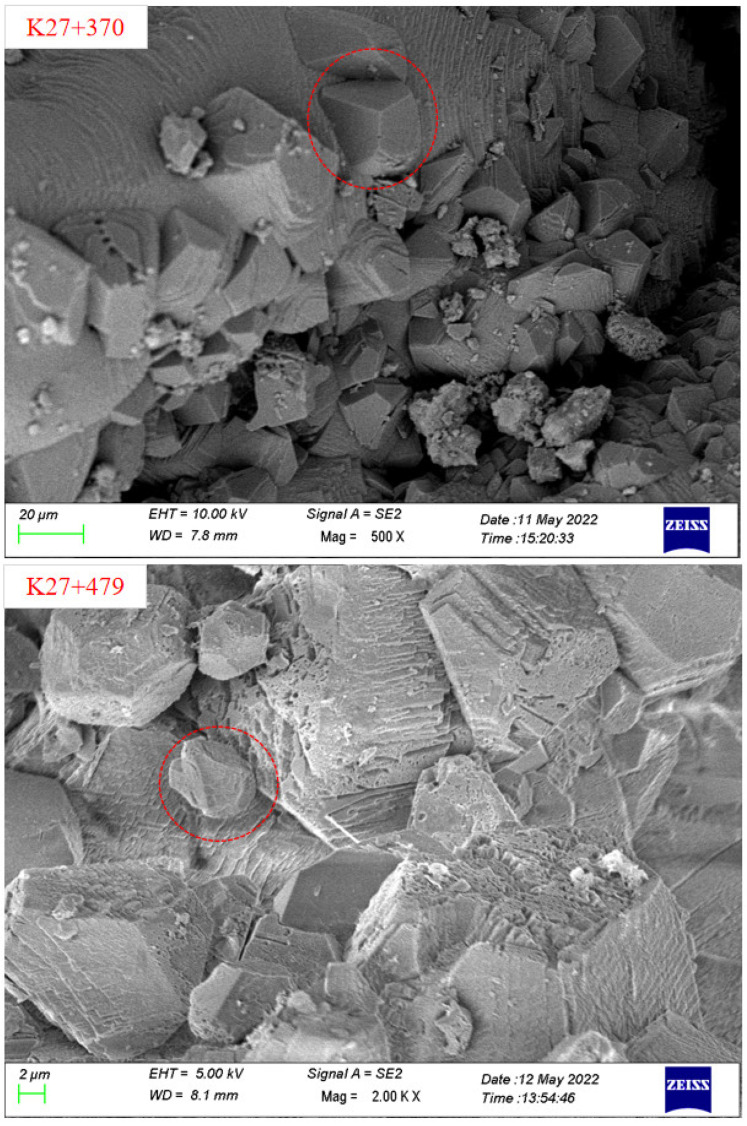

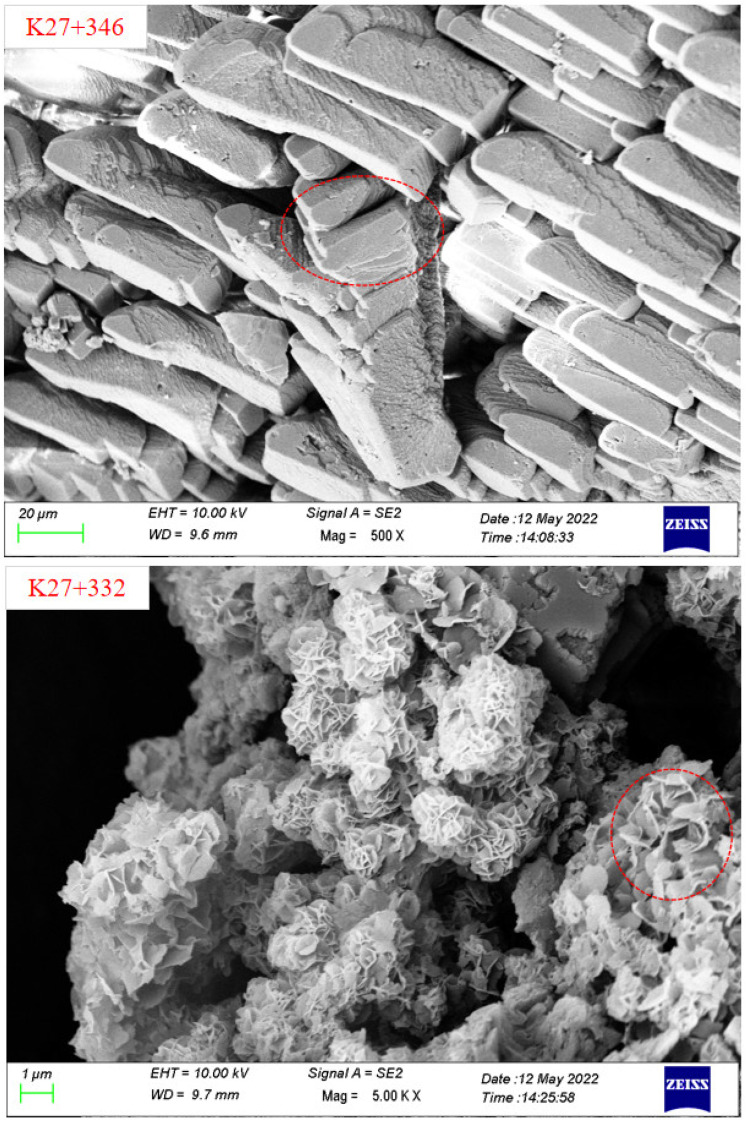

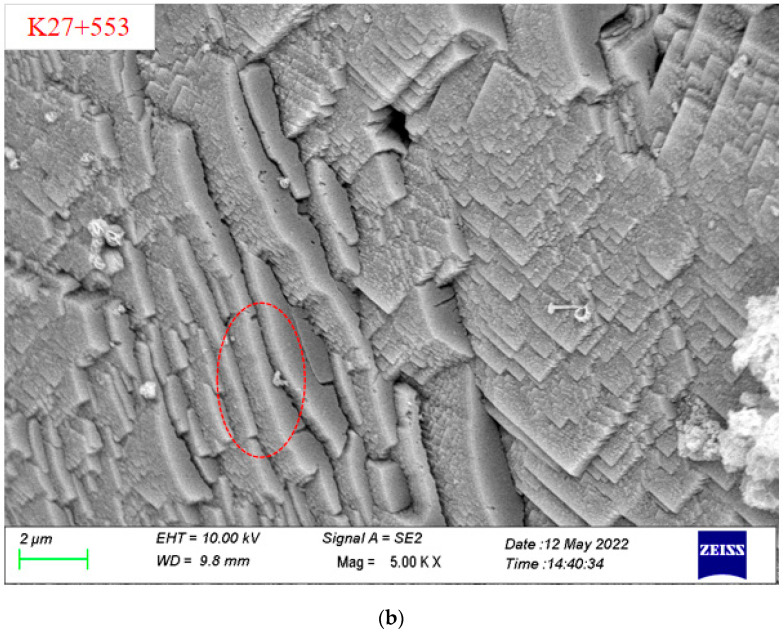
XRD diffraction pattern and micro morphology of crystal. (**a**) XRD diffraction pattern; (**b**) micro morphology of crystal.

The analysis of crystalline composition and microscopic forming were performed by XRD and SEM. The microscopic test of crystal samples was commissioned to Chongqing Nuojiang Two-dimensional Materials Research Institute Co., Ltd. The main compositions of the crystalline samples were determined by comparing the diffraction patterns of each crystalline material with the standard diffraction pattern of calcium carbonate, to compare their respective diffraction angles and peaks. Lee et al. [25] and Higashitani et al. [26] point out that CaCO_3_ crystals can be divided into calcite, aragonite, and vaterite, among which calcite is the easiest to form and the most common. Nebel and Epple [27] found that the XRD diffraction pattern of calcite demonstrated the main peak at the diffraction angle of 2*θ* = 29°, while the aragonite’s peaks were 2*θ* = 26° and 2*θ* = 46°. Figure 4 illustrates that the XRD diffraction patterns of crystalline samples all displayed the highest diffraction intensity at 2*θ* = 29°, indicating that the crystalline material was mainly calcium carbonate. According to SEM analysis of the crystalline material, most of the crystals were lumps, some crystals contain crusts, and the lumps were either tightly packed or loosely packed, indicating that the growth variation of crystalline material crystal shape was highly related to groundwater environment or surrounding temperature and humidity. This is consistent with the research results of Rinder et al. [28], Zhang et al. [29], Jiang et al. [30], Wu et al. [31], Stefanie et al. [32], Guo [33], and Ye et al. [34]. Calcium carbonate crystal plugging is mainly affected by pH value, temperature, CO_2_ partial pressure, water flow rate, drainage pipe slope, etc. The precipitation rate of calcite crystalline calcium carbonate is the highest when the pH value is close to 10 [33]. The natural calcium bicarbonate solution only produces calcite at 25 °C. The rapid evaporation and slow diffusion of the solution are conducive to the formation of aragonite [35]. When the water temperature is 40~50 °C, the crystal morphology is mostly regular blocky crystals, with the largest crystal size [36]. The partial pressure of CO_2_ has a great relationship with carbonate crystallization sedimentation, and CO_3_^2^− is mainly derived from CO_2_ dissolved in groundwater [31]. Liquid velocity, liquid level height, drainage pipe diameter, and the internal wall friction coefficient will also have an important impact on the precipitation crystallization rate of drainage pipes [37,38,39]. The precipitation of calcium carbonate depends not only on the hydraulic conductivity and CaCO_3_ content of surrounding rock, but also on the properties of lining materials and the geometry of the tunnel [40]. A low dosage of sodium aluminate will accelerate the leaching of calcium, thus reducing the leaching resistance of shotcrete. A high dosage of sodium aluminate will inhibit the leaching of calcium, thus improving the leaching resistance of shotcrete [41].

### 3.4. Fault Tree Model Construction and Analysis for Blocked Transverse Drainage Pipe

#### 3.4.1. Event Determination of Fault Tree Layers

Based on the theory of fault tree analysis model construction, a fault tree of the blocked transverse drainage pipe in Chongqing subway tunnel was constructed in the order of top event→logic gate→intermediate event→logic gate→bottom event.

(1) The top event was the blockage of transverse drainage pipe in the subway tunnel. The groundwater behind the tunnel lining was not only discharged from the tunnel through the longitudinal drainage pipe, but also discharged into the drainage ditches on both sides of the tunnel bed through the transverse drainage pipe. It was difficult to observe the longitudinal drainage pipe during tunnel operation even with other auxiliary methods, so the transverse drainage pipe became a very important event to judge the groundwater behind the tunnel lining. Therefore, the blockage of the transverse drainage pipe was identified as a top event in the construction of the fault tree.

(2) The intermediate events mainly included design and construction factors in engineering, groundwater, and surrounding rock factors in the geological environment, and atmospheric rainfall and microorganism factors in the ecological environment.

(3) Bottom event.

In terms of design, national and industry standards have specified the material and size, transverse slope, and installation spacing of tunnel transverse drainage pipe. Under normal construction, the design of transverse drainage pipe can meet the needs of tunnel operation, and thus the design factors were not considered in the construction of the fault tree.

In terms of construction, defects such as too small of a transverse drainage pipe slope, a flattened drainage pipe, drainage pipe damage, and concrete slurry flowing into the drainage pipe, can indirectly lead to the blockage of drainage pipes.

In terms of geological environment, the cation and anion concentrations, pH, temperature, and CO_2_ concentration in groundwater can cause crystalline blockage in transverse drainage pipes. The CO_2_ concentration at the drainage pipe was not tested during the survey, thus this factor was not considered in the model construction.

In terms of ecological environment, atmospheric rainfall brings a large amount of groundwater to the tunnel drainage system, and the presence of groundwater is what makes it possible for crystallization and blockage to occur in the drainage pipe, which basically does not exist if there is no groundwater. The surrounding environment affects the distribution of microorganisms in the groundwater, which further affects the survival of microorganisms in the drainage pipe. The groundwater development of the tunnel was investigated. There was a certain amount of flowing groundwater in the drainage pipe for a long time, and there was no microbial influence on the pipe blocking problem; thus the ecological factor was not considered in the construction of the fault tree.

#### 3.4.2. Construction of a Fault Tree

**Figure 5:**
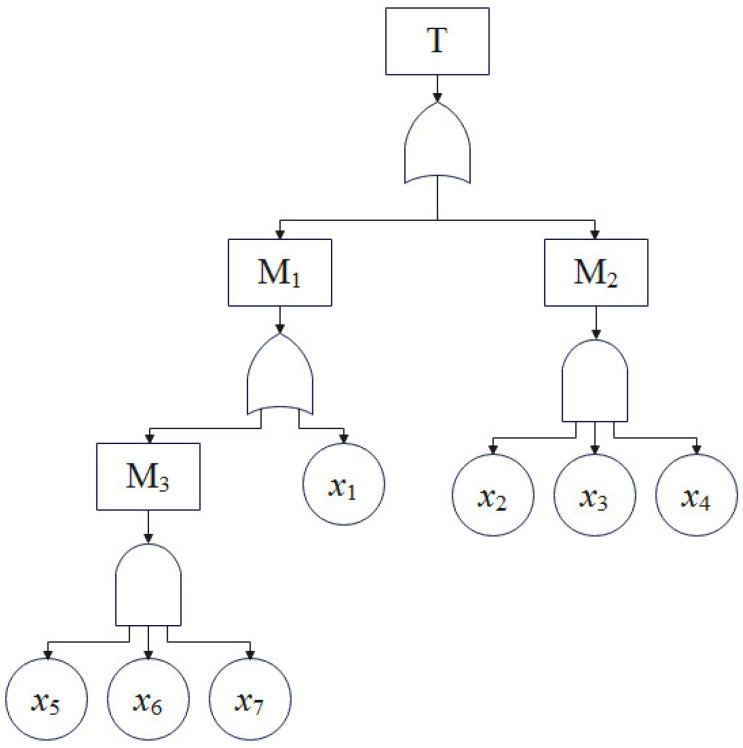
Fault tree model for blocking of horizontal drainage pipe of Metro Tunnel.

After determining the events involved in transverse drainage pipe blockage, a fault tree was established based on Heinrich’s Law, a theory about accident causality, as illustrated in Figure 5. The code factors are provied in Table 3. Through the construction of the fault tree, the subway tunnel operation managers can fully grasp all the possible causes (basic events *x*_1_~*x*_7_) of the top event (T), but the logical relationships between these basic events are not yet clear. Thus, qualitative and quantitative analysis of the fault tree was needed.

#### 3.4.3. Qualitative Analysis of the Fault Tree

The purpose of FTA is to discern the importance of the impact of the basic events on top events, (i.e., which of the many factors (basic events) that induce the occurrence of an accident (top event) are important and which are minor), so that the managers can take corresponding management countermeasures. The cut set is a set of several events in the fault tree. If these basic events occur, the top event is bound to happen. A minimum cut set is a cut set whose number of basic events cannot be reduced further, and a minimum cut set represents a failure mode that causes the top event of the fault tree to occur.

We used the up method to find the minimum cut set:

(1) $$M_{3}=x_{5}\cap x_{6}\cap x_{7}=x_{5}x_{6}x_{7}$$

(2) $$M_{2}=x_{2}\cap x_{3}\cap x_{4}=x_{2}x_{3}x_{4}$$

(3) $$M_{1}=M_{3}\cup x_{1}=x_{5}\cap x_{6}\cap x_{7}\cup x_{1}=x_{5}x_{6}x_{7}+x_{1}$$

(4) $$T=M_{1}\cup M_{2}=(x_{5}\cap x_{6}\cap x_{7})\cup x_{1}\cup (x_{2}\cap x_{3}\cap x_{4})$$

(5) $$\mathrm{Namely}\;T=x_{1}+x_{2}x_{3}x_{4}+x_{5}x_{6}x_{7}$$

There were 3 set terms in Equation (5), and 3 minimum cut sets are obtained: $G_{1}=\left\{x_{1}\right\},\;G_{2}=\left\{x_{2}x_{3}x_{4}\right\},\;G_{3}=\left\{x_{5}x_{6}x_{7}\right\}$.

By analyzing the minimum cut set of top events, the subway tunnel operation managers can understand the logical relationship combination of all the basic events that cause top events to occur.

#### 3.4.4. Quantitative Analysis of Fault Tree

The basic events in the fault tree are not equally important. Some basic events or combinations of them cause a top event as soon as they occur, while others do not. Qualitative and quantitative analysis of the importance of each basic event in the fault tree can be very valuable to subway tunnel operation managers for formulation of effective management strategies or implementation of treatment measures. The probability of occurrence of each basic event was calculated separately by conducting a statistical analysis of the interdistrict tunnel site survey, data review, and communication with the managers, as illustrated in Table 4.

1. Importance Analysis of Basic Event Structure

The basic numbers contained in the minimum cut set of the fault tree are not equal, and the structural importance of each basic event can be approximately discriminated and compared by Formula (6):

(6) $$I_{\varphi }\left(j\right)=\underset{x_{j}\in G_{r}}{\sum }\frac{1}{2^{n_{j}-1}}$$

where: $I_{\varphi }\left(j\right)$ is the basic event $x_{j}$ approximate discriminant value of structural importance, the greater the value of $I_{\varphi }\left(j\right)$, the greater the importance, $x_{j}\in G_{r}$ is the basic event $x_{j}$ belongs to the minimum cut set $G_{r}$, and $n_{j}$ is the number of basic events contained in the minimum cut set where the basic event $x_{j}$ is located.

The importance of each event structure was judged as follows: $I_{\varphi }\left(1\right)=\sum_{x_{j}\in G_{r}}\frac{1}{2^{n_{j}-1}}=\frac{1}{2^{1-1}}=1.00$.

By the same token $I_{\varphi }\left(2\right)=I_{\varphi }\left(3\right)=I_{\varphi }\left(4\right)=\sum_{x_{j}\in G_{r}}\frac{1}{2^{n_{j}-1}}=\frac{1}{2^{3-1}}=0.25$; $I_{\varphi }\left(5\right)=I_{\varphi }\left(6\right)=I_{\varphi }\left(7\right)=\sum_{x_{j}\in G_{r}}\frac{1}{2^{n_{j}-1}}=\frac{1}{2^{3-1}}=0.25$.

According to the calculation results, the structural importance coefficients of each basic event are arranged as follows: $I_{\varphi }\left(1\right)>I_{\varphi }\left(2\right)=I_{\varphi }\left(3\right)=I_{\varphi }\left(4\right)=I_{\varphi }\left(5\right)=I_{\varphi }\left(6\right)=I_{\varphi }\left(7\right)$.

That is, on the fault tree structure, the basic event $x_{1}$ has the greatest influence on the top event, followed by $x_{2},x_{3},x_{4},x_{5},x_{6},\;\mathrm{and}\;x_{7}$. This finding demonstrates that if the transverse drainage pipe of the tunnel is filled with the lining concrete slurry during the construction process, the transverse drainage pipe will inevitably be blocked, while other factors of the drainage pipe caused by the construction have relatively small influence.

2. Probability Importance Analysis of Basic Events

Since the occurrence probability function *g* of the top event is a multiple linear function, the probability importance coefficient of the basic event can be obtained as long as the partial derivative of the independent variable *q_i_* is obtained once; that is:

(7) $$I_{g}=\frac{\partial g}{\partial q_{i}}$$

The probability of occurrence of the top event can be approximately calculated using Formula (8), namely:

(8) $$g\approx \overset{N_{G}}{\underset{r=1}{\sum }}\prod_{x_{i}\in q_{i}}q_{i}$$

where: $N_{G}$ is the minimum number of cut sets, *r* is the ordinal number of the minimum cut set, *i* is the basic event ordinal, and *q_i_* is the probability of occurrence of the *i*th basic event.

In combination with the basic event probability in Table 4, the occurrence probability of the top event can be calculated by Formula (8), $g=q_{1}+q_{2}q_{3}q_{4}+q_{5}q_{6}q_{7}\approx 0.045$.

Use Equation (7) to calculate the probability importance coefficient of basic events: $I_{g}\left(1\right)=\frac{\partial g}{\partial q_{1}}=1$.

By the same token, $I_{g}\left(2\right)=q_{3}q_{4}=0.010$; $I_{g}\left(3\right)=q_{2}q_{4}=0.028$; $I_{g}\left(4\right)=q_{2}q_{3}=0.042$; $I_{g}\left(5\right)=q_{6}q_{7}=0.000$; $I_{g}\left(6\right)=q_{5}q_{7}=0.001$; $I_{g}\left(7\right)=q_{5}q_{6}=0.001$.

According to the above calculation results, the probability importance coefficient of each basic event is arranged as follows: $I_{g}\left(1\right)>I_{g}\left(4\right)>I_{g}\left(3\right)>I_{g}\left(2\right)>I_{g}\left(6\right)=I_{g}\left(7\right)>I_{g}\left(5\right)$.

That is, reducing the basic event $x_{1}$ can rapidly reduce the occurrence probability of the top event, followed by $x_{2},x_{3},\;\mathrm{and}\;x_{4}$; the least sensitive is the basic event $x_{5}$.

3. Critical Importance Analysis of Basic Events

The critical importance is a standard for measuring the importance of basic events from the perspective of sensitivity and occurrence probability. The relationship between the critical and probability importance is:

(9) $$I_{G}\left(i\right)=\frac{q_{i}}{g}I_{g}\left(i\right)$$

In combination with the probability importance coefficient of basic events calculated previously, the importance coefficient of basic events is: $I_{G}\left(1\right)=\frac{q_{i}}{g}I_{g}\left(i\right)=\frac{0.0417}{0.045}\times 1\approx 0.9227$.

By the same token, $I_{G}\left(2\right)=I_{G}\left(3\right)=I_{G}\left(4\right)\approx 0.0769$; $I_{G}\left(5\right)=I_{G}\left(6\right)=I_{G}\left(7\right)\approx 0.0004$.

According to the above calculation results, the critical importance coefficient of each basic event is arranged as follows: $I_{G}\left(1\right)>I_{G}\left(2\right)=I_{G}\left(3\right)=I_{G}\left(4\right)>I_{G}\left(5\right)=I_{G}\left(6\right)=I_{G}\left(7\right)$.

Compared with the probability importance, the importance of the basic events $x_{2}$ and $x_{3}$ increases, because of $x_{2},x_{3}$. The two basic events play a direct role in the occurrence of crystal blockage in the horizontal drainage pipe, and the probability of their occurrence is relatively large.

4. Summary and Analysis of the Importance of Basic Events

The structural, probability, and critical importance of each basic event are provided in Table 5.

As can be observed in Table 5, from the analysis of structural importance, the basic event $x_{1}$ is the most important in the fault tree, thus the managers should pay more attention to it in system construction; from the probability importance analysis, the basic events $x_{1}$, $x_{4}$, $x_{3}$, and $x_{2}$ have the greatest impact on the occurrence probability of the top events, thus the managers should strengthen supervision and monitoring of them so as to minimize their occurrence probability; from the critical importance analysis, the basic event $x_{1}$ was the most likely event to cause top events, followed by the basic events $x_{4}$, $x_{3}$, and $x_{2}$, thus the managers should pay special attention to controlling these events in particular.

## 4. Analysis of Countermeasures for the Maintenance of Transverse Drainage Pipes in Subway Tunnels

Although the maintenance of a drainage system is mainly performed during tunnel operation, the failure to timely and effectively address the drainage system blockage that occurred during tunnel construction will result in the additional cost of drainage system maintenance during tunnel operation. Therefore, the maintenance countermeasures of drainage systems should be started during tunnel construction and gradually extended to tunnel operation.

### 4.1. Preventive Maintenance of Transverse Drainage Pipe during Tunnel Construction

FTA demonstrates that during drainage pipe construction, the pouring of the secondary lining makes it easy for the concrete slurry to flow into the drainage pipe, and if it is not unblocked in time, the concrete slurry will solidify on the inner wall of the drainage pipe, which will aggravate the blockage of the drainage pipe. According to the Railway Tunnel Construction Quality Acceptance Criteria (TB10417-2018), the structural form, structural elevation, and longitudinal slope of drainage ditch (pipe) structures in tunnels shall meet the design requirements and shall be inspected through measurement. The location, spacing, size, and slope of transverse drainage pipes shall meet the design requirements, and the drainage of the blind pipe, ditch, hole, and groove shall be smooth and free from blockage. The inspection methods are mainly observation, measurement, and image data retention; there is no relevant requirement on the dredging of concrete slurry or other debris in drainage pipes, which undoubtedly lays hidden danger for the smooth drainage of drainage pipe.

In addition to the concrete slurry flowing into the drainage pipe during tunnel construction, in recent years, crystalline blockage in drainage pipes has also emerged during tunnel construction, whose main influencing factors include the basic events $x_{2}$, $x_{3}$, and $x_{4}$, demonstrating that the crystalline blockage in drainage pipe appears during tunnel construction and extends to tunnel operation. Therefore, preventive maintenance of tunnel drainage systems during tunnel construction can be performed from the following aspects.

(1) Construction Quality Control of Transverse Drainage Pipe in Subway Tunnel

Usually, the transverse drainage pipe in subway tunnel is short (1~1.5 m), so its installation spacing and slope can strictly meet the design requirements during construction. After the pouring of lining concrete, check the presence of concrete slurry in drainage pipes one by one. Due to the normally long tunnel construction cycle, the crystalline blockage in drainage pipe appears during tunnel construction. As time increases, the crystalline materials gradually adhere to and are closely bonded with the pipe wall, and cannot be effectively removed by general dredging measures.

**Figure 6:**
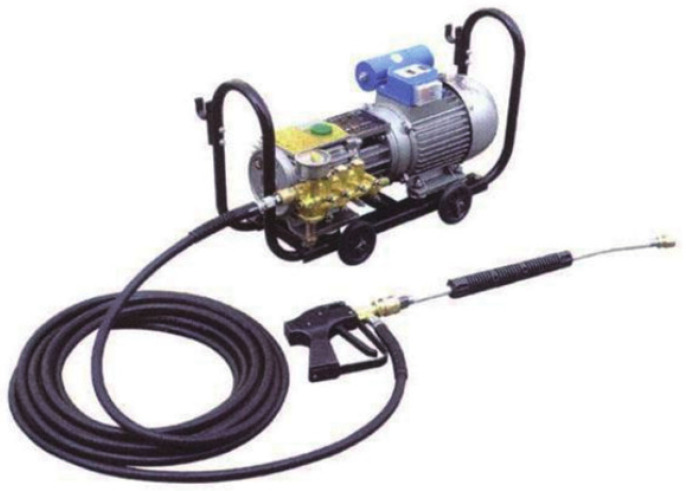
High pressure water jet equipment.

**Figure 7:**
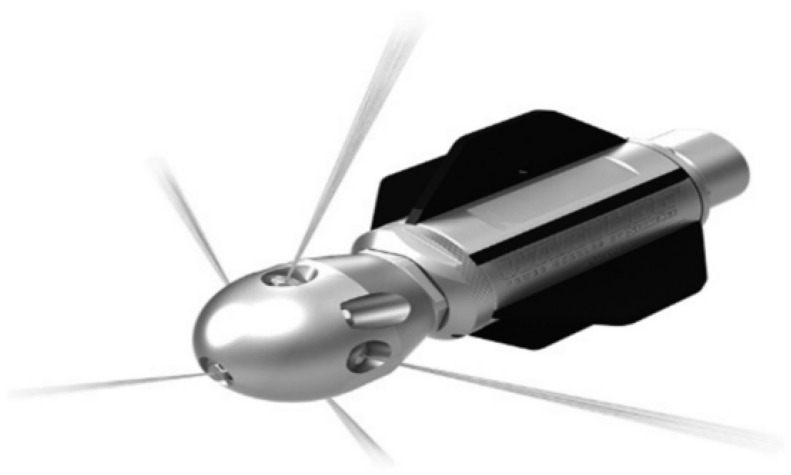
Water jet nozzle.

The concrete slurry or crystalline blockage in drainage pipes during tunnel construction can be dredged by the mechanical method, which is the earliest and most common pipe dredging method. According to the dredging mechanism, it can be divided into two categories. One is to use high pressure equipment to produce a strong water flow to scour off or pulverize the blockage on the pipe wall, at the same time producing a stirring force to suspend and discharge the scaling matter out of the pipe. An example is the high pressure water jet method. As illustrated in Figure 6 (high pressure water jet equipment) and Figure 7 (water jet nozzle), the water flow is pressurized into the water jet nozzle, and then the reaction force generated by the jet pushes the nozzle and hose forward together to scour off the crystalline material adhering to the pipe wall, cleaning the pipe wall [42,43,44,45]. With high removal efficiency, low economic cost, no environmental pollution, and good adaptability of pipe, this method has a good removal effect on early soft slurry or crystals in the drainage pipe. However, it is not applicable to the drainage pipe with longtime crystallization, or hard and dense concrete slurry or scale. Therefore, the blocked drainage pipe should be flushed in a timely manner during tunnel construction.

**Figure 8:**
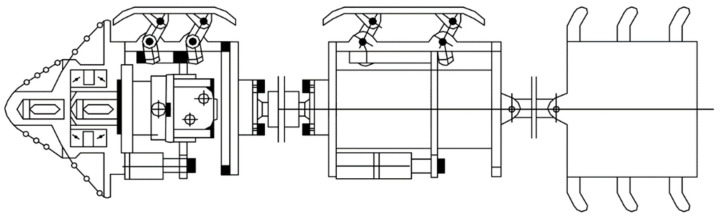
Pipeline dredging robot.

The other is to use the scraper designed according to the pipe size to rub and cut crystals, to forcibly break and loosen the crystalline deposits, so as to dredge the blocked drainage pipe. As illustrated in Figure 8, the head of the pipe dredging robot is equipped with cleaning devices, such as cutting tools and steel wire brushes, which can break and loosen the crystalline deposits in the drainage pipe. The crystalline deposits are then washed away along the pipe water flow or an additional water flow in the direction of the progress of the robot [46]. With high degree of automation, good dredging quality, reliable performance, and convenient operation, this method is applicable to the drainage pipe with long blocked sections and large blockage hardness. However, it may damage the drainage pipe when the drainage pipe has been squeezed and has undergone large deformation; it is not commonly used in practice due to the complex equipment and high cleaning costs.

(2) Anti-Crystallization Blockage Drainage Pipe

Anti-crystallization blockage drainage pipe is mainly used to prevent crystalline blockage in tunnel drainage pipes. In order to prevent crystalline blockage in tunnel drainage pipes, the most direct approach is to prevent the deposition of crystals on the drainage pipe, so that even corrosive media inducing crystallization in groundwater will not form deposits in the drainage pipe to cause blockage. Jiang et al. [47] proposed a protective coating method to reduce the deposition of crystals on the pipe wall by using coatings to hydrophobically treat the water pipe wall, so that a hydrophobic force is formed between the crystalline solution and the water pipe coating to reduce the adhesion capacity of crystals. Liu et al. [48] and Liu et al. [49] proposed that flocking PVC tunnel drainage pipe can inhibit the adhesion of crystals to the pipe wall, and the use of thermally conductive tunnel drainage pipe can avoid the crystalline blockage in tunnel drainage pipes because of the low temperature. Chen [50] proposed that in order to reduce the kinetic energy loss of water in the flow process and improve the drainage capacity of a tunnel drainage system, the angle of drainage pipe connection and the friction resistance of drainage pipe material can be reduced, and the radius of curvature can be adjusted, so that the crystal precipitation can be reduced as a consequence.

### 4.2. Preventive Maintenance of Transverse Drainage Pipe during Tunnel Operation

The results of the field investigation and FTA demonstrated that, in addition to the disease of drainage pipe during tunnel construction, the crystalline blockage in drainage pipe occurred most frequently during tunnel operation. The main factors involved in drainage pipe crystallization blockage include the basic events $x_{2}$, $x_{3}$, and $x_{4}$. During tunnel operation, long-term monitoring of the above three main factors should be performed, and treatment of crystals can be guided according to the change law of monitoring data.

(1) Monitoring of Groundwater Ion Concentration

According to the Surin Classification, the groundwater is divided into four categories: calcium chloride (CaCl_2_) type, sodium sulfate (Na_2_SO_4_) type, magnesium chloride (MgCl_2_) type, and sodium bicarbonate (NaHCO_3_) type. The bicarbonate type groundwater is rich in HCO^−^, which will combine with free calcium and directly precipitate with cement hydration products when the groundwater passes through the tunnel primary support shotcrete. The reaction equation is

Ca(HCO_3_)_2_ + Ca(OH)_2_ = 2CaCO_3_↓ + 2H_2_O (10)

This process will also accelerate the erosion process of the groundwater on shotcrete, resulting in an increase of concrete porosity and the alkali-aggregate reaction of concrete [51,52,53]. The karst type groundwater is rich in Ca^2+^ and Mg^2+^, which directly provides a material source for crystallization in the drainage pipe and aggravates the crystalline blockage.

Moreover, the groundwater inevitably scours, permeates, and dissolves the surrounding rock in the process of long-term seepage. The resulting CO_3_^2−^ and Ca^2+^ from the dissolution of soluble carbonate (such as limestone, dolomite, etc.) will permeate into the concrete with the flow of groundwater, which promotes the calcium precipitation and dissolution process in concrete. HCO^−^ directly combines with free Ca^2+^ in concrete to form CaCO_3_, which is brought into the drainage pipe by seepage water to form blockage. Due to the water pressure, the water surrounding rock in the water-rich tunnels often seeps out through the concrete fissure channels, composed of concrete pores and cement hydration channels. In the process of continuous seepage of groundwater, the shotcrete fissure channels are constantly widened and enlarged. The more fissures and macroscopic cracks produced, the more calcium loss in the tunnel shotcrete. Also, the shotcrete has a high calcium content due to the high cement content. Therefore, as long as the groundwater continuously flows out of the shotcrete surface or the fissures, the free calcium produced by cement hydration will be continuously carried out of the concrete and gradually deposited on the tunnel drainage pipe to form blockage.

**Figure 9:**
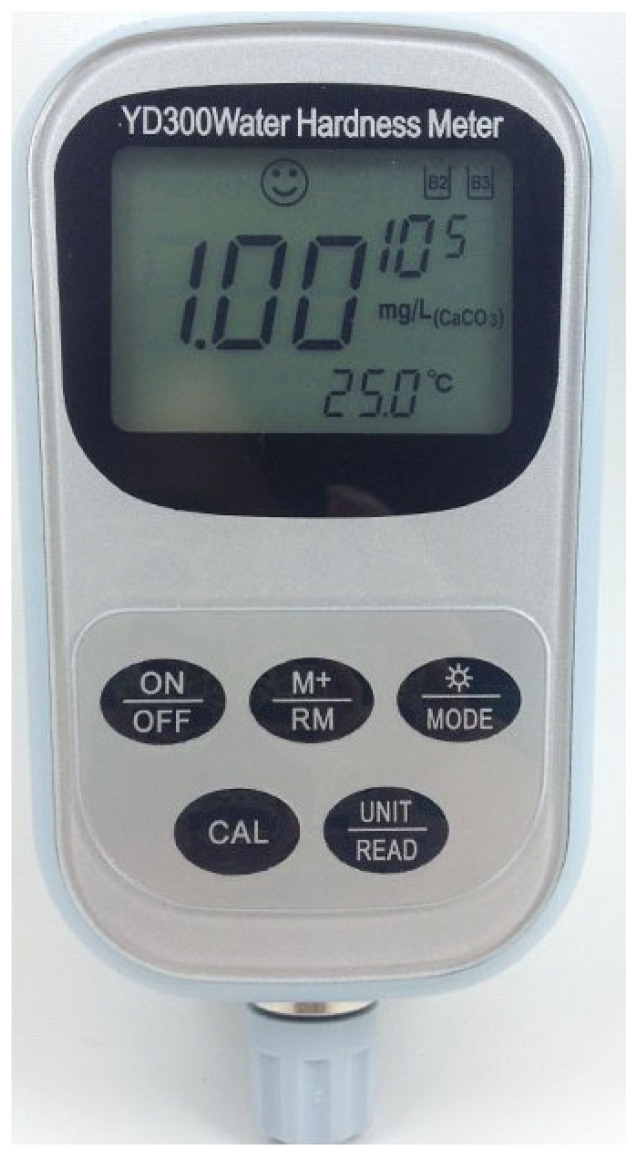
Portable groundwater calcium ion concentration detector.

**Figure 10:**
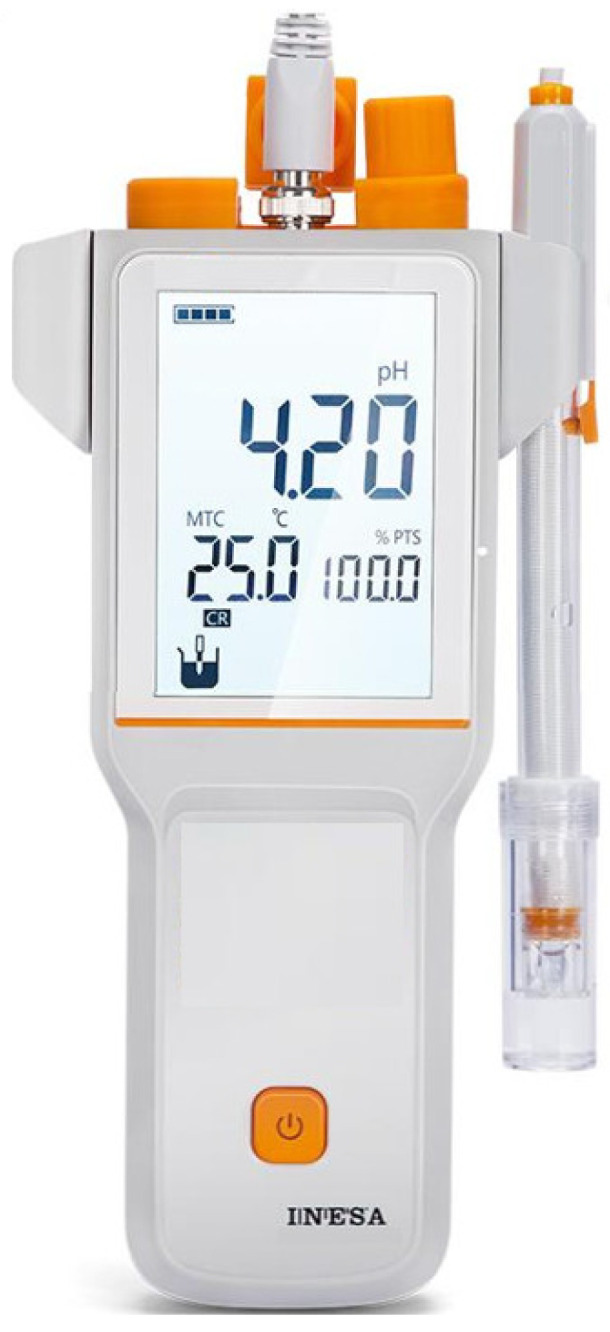
Portable groundwater pH value and groundwater temperature monitor.

As can be observed from the above analysis, the ion concentration in groundwater is relatively changing, and it is closely related to the physicochemical effects of groundwater on the tunnel support structure, especially during the first few years of tunnel construction and operation. Thus, the managers should pay special attention to the change of ion concentration in this period of time. A portable ion concentration meter (Figure 9) can be used for testing or the groundwater samples collected on the site can be taken back to the room for testing. The crystalline composition and chemical reaction equation (Figure 10) demonstrates that the crystalline cations in the drainage pipe are mainly Ca^2+^; thus Ca^2+^ should be mainly monitored in the monitoring of groundwater ion concentration.

(2) Monitoring of Groundwater pH

The study demonstrates that the water filling state and pH have a coupling effect on the crystallization amount. The crystallization amount of the pipe increases with the increase of pH. When the pH is 8~10, the crystallization amount of the semi-filled state is higher [54], and the precipitation rate of calcite calcium carbonate is the highest when the pH is close to 10 [33]. The site conditions and test results illustrate that the groundwater at the blockage location of the drainage pipe is strongly alkaline. Thus, long-term monitoring of groundwater pH is of great significance in guiding the crystallization treatment of the tunnel drainage pipe. The groundwater pH can be measured by a portable pH meter (Figure 10), which can be carried by operation managers during daily or regular inspections to timely measure the groundwater pH at the crystalline blockage location of the drainage pipe, so as to provide a basis for the maintenance of the drainage pipe.

(3) Monitoring of Groundwater Temperature

Crystal blockages in tunnel drainage pipes are products of the chemical reaction of cations and anions in groundwater. In the vast majority of cases, the temperature has a certain effect on promoting the chemical reaction. Studies have demonstrated that a natural calcium bicarbonate solution only produces calcite at 25 °C, and the rapid evaporation and slow diffusion of the solution is conducive to the generation of aragonite [35]. When the groundwater temperature is at 40~50 °C, the crystals formed are mostly regular block crystals with the largest size [36]. Thus, the groundwater temperature has a significant effect on the formation of calcium carbonate crystals, which is related to the difficulty of treating calcium carbonate crystals. Therefore, the subway tunnel operation managers can carry a portable groundwater temperature thermometer (Figure 10) around during daily or regular inspection to timely measure groundwater temperature, so as to provide a basis for the maintenance of drainage pipes.

The above analysis demonstrates that the groundwater ion concentration, pH value, and temperature around the tunnel change at any time. Therefore, the subway tunnel operation managers can sample the groundwater at the drainage pipe prone to crystal blockage during daily inspection, and test it with a portable detector. Through the indoor test analysis, they can master the change law of groundwater ion concentration with seasons, so as to formulate treatment frequencies and methods for the crystal blockage in drainage pipes.

### 4.3. Maintenance and Treatment of Transverse Drainage Pipe during Tunnel Operation

In terms of operation and maintenance of subway tunnel drainage system, the aforementioned diseases during tunnel construction can also be treated by a mechanical method, in addition to physical or chemical techniques.

(1) Physical Treatment Techniques

Ultrasonic treatment is a physical treatment technique that can now be used for the treatment of transverse drainage pipes or drainage ditches in subway tunnels. A transducer is used to emit ultrasonic waves, which directly act on the contact between the crystals and pipe wall through acoustic vibration and cavitation effect, so as to remove the crystals. Ultrasonic waves can crush large crystals in the pipe, while the frequency and intensity of the ultrasonic wave, as well as the type, temperature, concentration and pH of the solution, are all related to the efficiency of the ultrasonic removal of crystals. The combination of different parameters of ultrasonic waves is essential to improve the removal of calcium carbonate crystals [55,56].

**Figure 11:**
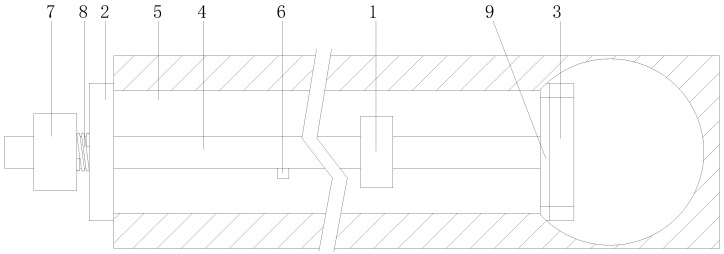
Schematic diagram of ultrasonic resonance treatment device for crystal blockage of transverse drainage pipe of Metro Tunnel.

In view of the crystalline blockage in transverse drainage pipes in subway tunnels, the following ultrasonic resonance treatment device (Figure 11) is designed. The device mainly includes an ultrasonic resonance part, an external blocking plate, internal retractable blocking components, and a water injection pipe. Both the external blocking plate and the internal retractable blocking component are installed on the water injection pipe. The external blocking plate blocks the outlet end of the transverse drainage pipe, and the internal retractable blocking component blocks the inlet end of the transverse drainage pipe. The ultrasonic resonance part is installed on the water injection pipe with an outlet between the external blocking plate and the internal retractable blocking component. In Figure 11, 1 is the ultrasonic resonance part, 2 is the external blocking plate, 3 is the internal retractable blocking component, 4 is the water injection pipe, 5 is the transverse drainage pipe, 6 is the outlet, 7 is the controller, 8 is the first offset spring, and 9 is the arc-shaped rubber sealing gasket.

(2) Chemical Treatment Techniques

Chemical treatment is to inject a dissolving agent to react with crystals in the drainage pipe, so that crystals will gradually dissolve and flow away along the pipe water flow. The chemical method is simple, effective, and economically efficient, but it causes corrosive damage to the tunnel structure and has a great impact on the surrounding groundwater and soil environment. Thus, it is used less in the practical treatment of crystalline blockages in tunnel drainage pipes. At present, the commonly used chemical treatment methods are complexation, acid, and synthetic methods. The chemical treatment technique is convenient, where the chemical reagents can be directly poured into the drainage ditch, or injected into the transverse drainage pipe through special equipment, so as to achieve the treatment of crystalline blockages in tunnel drainage pipes.

## 5. Conclusions

The influencing factors of crystalline blockage in transverse drainage pipes in subway tunnels mainly include design and construction factors in engineering, groundwater, and surrounding rock factors in the geological environment, and atmospheric rainfall and microbial factors in the ecological environment. More than 95% of the formerly designed transverse drainage pipes of subway tunnel in the study area have lost their drainage function due to construction or other factors, and more than 80% of the drainage holes added during tunnel operation were blocked by crystallization. Therefore, the drainage pipe blockage must be highly emphasized during tunnel design, construction, and operation.

1. The Ca^2+^ content in groundwater is lower than 100 mg/L, and the HCO_3_^−^ content is in the range of 100~220 mg/L; most of the groundwater is strongly alkaline with pH greater than 8; the groundwater temperature is in the range of 20 ± 3 °C.
2. The basic events of transverse drainage pipe blockage constitute 3 minimal cut sets of the fault tree, and the basic events $x_{1}$, $x_{4}$, $x_{3}$, and $x_{2}$ are the main causal factors of transverse drainage pipe blockage.
3. The preventive maintenance of transverse drainage pipes during tunnel construction includes quality control of the drainage pipes and application of anti-crystallization blockage drainage pipes; the preventive maintenance of transverse drainage pipes during tunnel operation includes the monitoring of groundwater ion concentration, pH, and temperature; the treatment of transverse drainage pipes during tunnel operation includes physical treatment techniques, such as ultrasonic resonance, and chemical treatment techniques, such as acid-base neutralization reactions.

## Figures and Tables

**Table 1:** Site sampling locations and tests.

Sample Type	Sampling Station	Groundwater pH	Groundwater Temperature	Remarks
Groundwater sample	K27 + 338	9.51	17.80	1.5 L
	K27 + 352	9.00	19.50	1.5 L
	K27 + 428	10.90	22.10	1.5 L
	K27 + 450	7.71	21.40	1.5 L
	K27 + 479	7.84	20.20	1.5 L
	K27 + 553	8.87	23.10	1.5 L
	K28 + 070	8.49	19.60	1.5 L
Crystal sample	K27 + 370	10.38	19.20	/
	K27 + 479	7.84	20.20	/
	K27 + 346	10.40	18.50	/
	K27 + 332	10.62	16.00	/
	K27 + 553	8.87	23.10	/

**Table 2:** Statistics of groundwater test and analysis results.

Groundwater Analysis Project		Station Number						
		K27 + 338	K27 + 352	K27 + 428	K27 + 450	K27 + 479	K27 + 553	K28 + 070
Groundwater ion concentration (mg/L)	K^+^	8.20	7.02	12.4	6.07	6.23	9.13	10.6
	Na^+^	45.8	37.6	42.7	39.9	36.0	57.9	44.3
	Ca^2+^	45.0	52.9	76.8	79.4	68.3	38.6	54.7
	Mg^2+^	9.75	9.39	0.0	15.5	15.4	11.2	16.8
	Cl^−^	55.1	37.5	33.1	61.2	39.7	46.3	46.3
	SO_4_^2−^	108	105	81.7	82.2	89.3	82.6	89.3
	HCO_3_^−^	100	118	0.0	218	187	137	172
	CO_3_^2−^	0	0	21.5	0	0	0	3.07
	OH^−^	0	0	40.9	0	0	0	0
Total hardness (calculated by CaCO_3_) (mg/L)		153	171	192	262	234	143	206
Free CO_2_ (mg/L)		9.11	4.55	0	20.5	11.4	11.4	13.7
pH value	Indoor test	8.08	7.76	10.47	8.92	8.59	8.48	8.20
	Field test	9.51	9.00	10.90	7.71	7.84	8.87	8.49
Field test temperature (°C)		17.80	19.50	22.10	21.40	20.20	23.10	19.60

**Table 3:** Code factor statistics.

Code	Factor	Code	Factor
**T**	Blocking of cross drainage pipe of subway tunnel	***x*_3_**	Groundwater pH value
**M_1_**	Impact of drainage pipe construction	***x*_4_**	Groundwater temperature
**M_2_**	Influence of surrounding geological environment	***x*_5_**	Drainage pipe slope is too small
**M_3_**	Construction defects of drainage pipe	***x*_6_**	The drainage pipe was flattened
***x*_1_**	Concrete slurry enters the drainage pipe	***x*_7_**	Drainage pipe damaged
***x*_2_**	Concentration of anions and cations in groundwater	/	/

**Table 4:** Probability of occurrence of basic events.

Basic Events	Probability of Occurrence	Basic Events	Probability of Occurrence
** $x_{1}$ **	0.0417	$x_{5}$	0.0417
** $x_{2}$ **	0.3333	$x_{6}$	0.0208
** $x_{3}$ **	0.1250	$x_{7}$	0.0208
** $x_{4}$ **	0.0833		

**Table 5:** Importance of basic events.

Basic Events	Structural Importance	Probability Importance	Critical Importance
** $x_{1}$ **	1.00	1.000	0.9227
** $x_{2}$ **	0.25	0.010	0.0769
** $x_{3}$ **	0.25	0.028	0.0769
** $x_{4}$ **	0.25	0.042	0.0769
** $x_{5}$ **	0.25	0.000	0.0004
** $x_{6}$ **	0.25	0.001	0.0004
** $x_{7}$ **	0.25	0.001	0.0004

## Data Availability

The study did not report any data.

## Glossary

**Acronyms**	**Constituent Words**
FTA	Fault Tree Analysis
WBS-RBS	Work Breakdown Structure and Risk Decomposition Structure
T-S	Top-Second
TBM	Tunnel Boring Machine
ATS	Automatic Train Supervision
AHP	Analytic Hierarchy Process
XRD	X-Ray Diffraction
SEM	Scanning Electron Microscope
